# Novel markers of coronary inflammation in patients with type 2 diabetes

**DOI:** 10.1007/s12350-019-01866-x

**Published:** 2019-09-06

**Authors:** J. P. M. Andrews, M. R. Dweck

**Affiliations:** 1 British Heart Foundation, Centre for Cardiovascular Science, University of Edinburgh, Edinburgh, EH16 4SB UK; 2 Icahn School of Medicine at Mount Sinai, Translational and Molecular Imaging Institute, New York, USA

Atherosclerotic cardiovascular disease (CVD) is a leading cause of death in type 2 diabetics with myocardial infarction and stroke responsible for twice as many deaths in type 2 diabetics than non-diabetics. The primary mechanism responsible for myocardial infarction and stroke is atherosclerotic plaque rupture. Systemic inflammation is closely linked to atherosclerotic inflammation and increases cardiovascular event risk.^1^ Histologically, ruptured plaques display a metabolically active lipid cores with a large inflammatory component. Conventional plaque imaging methods such as ultrasound, invasive, CT, and MR angiography focus on morphological plaque features of vulnerability but cannot directly assess the metabolic activity of the plaque. 18F-FDG PET/CT is a hybrid imaging technique using a radiolabeled analog of glucose (18-Fluorine labeled to Fluorodeoxyglucose/18F-FDG) to provide anatomical (CT) and metabolic (PET) information on atherosclerotic plaque. Uptake of FDG has been shown in numerous studies to correlate closely with plaque macrophage burden. Recent large-scale studies suggest that modulation of systemic and atherosclerotic inflammation reduces cardiovascular events.^2^

In patients with type 2 diabetes, Pioglitazone (but not Glimepiride) reduces atherosclerotic inflammation as assessed by 18F-FDG PET/CT independent of its hypoglycaemic effect.^3^ Therefore, identification of those most likely to benefit from an anti-inflammatory response to pioglitazone is likely to be beneficial.

In a cohort study of 38 reasonably well-controlled type 2 diabetics on standard oral hypoglycaemic agents (OHAs), *Tahara et al*. aimed to identify clinical variables that predicted reduction of coronary inflammation after a 4-month treatment period with add-on Pioglitazone (*n* = 16) or Glimepiride (*n* = 22). At baseline and 4 months post add-on therapy, all patients underwent a 75 g oral glucose tolerance test (OGTT), fasting plasma glucose (FPG) and insulin, 30-min, 60-min, 90-min, and 120-min postload plasma glucose, serum levels of pigment epithelium-derived factor [PEDF (a novel marker of insulin resistance)], and an 18F-FDG PET/CT.

Coronary inflammation was assessed through calculation of standardized uptake values (SUV) within the left main trunk (LMT) and divided by the SUV within the superior vena cava to give a target-to-background ratio (TBR) of atherosclerotic plaque to blood pool uptake (LMT-TBR). As glucose is a competitive antagonist of FDG, serum levels affect cellular uptake therefore a correction was made for fasting plasma glucose (FPG) on the day on scanning.

Add-on therapy reduced fasting, 30-, 60-, 90-, and 120-minute postload glucose along with HBA1c levels and LMT-TBR. There was a significant and independent correlation of baseline non-use of aspirin (*p* = 0.03), baseline-elevated PEDF (*R*^2^ = −0.378, *p* = 0.019), Δ PEDF (*R*^2^ = 0.379, *p* = 0.016), and Δ120-min glucose (*R*^2^ = 0.436, *p* = 0.006) with an 11% reduction in overall mean LMT-TBR values (2.17 ± 0.53 to 1.93 ± 0.55). The authors suggest baseline PEDF, ΔPEDF and Δ120 min post prandial glucose and as potential therapeutic targets to reduce coronary inflammation in type 2 diabetics and that aspirin may reduce the anti-inflammatory effect of pioglitazone.

The study does, however, have limitations. LMT-TBR values were only significant reduced when corrected for FPG, which critically was not measured at the same time as the PET/CT. This confounds the interpretation of the data in 2 ways; firstly, FPG is influenced by diabetic medications therefore the measured SUVs could remain the same but the adjusted TBRs would change based on the FPG value. Secondly, patients with type 2 diabetes unsurprisingly have quite variable daily plasma glucose levels therefore plasma glucose at the time of the scan is highly likely to differ from the FPG used to correct LMT-SUV. Interestingly, uncorrected TBR values in the aorta and LMT were not significantly reduced by either add-on therapy (LMT-TBR 1.44 ± 0.31 vs 1.45 ± 0.34, Aorta-TBR 1.55 ± 0.29 vs 1.48 ± 0.27). The legitimacy of the authors results therefore heavily rely on the FPG at the time of the PET/CT scan (which we do not know) and the validity of the correction calculation.

Moreover, adjudication of FDG uptake within the coronary arteries and particularly the LMT is challenging.^4^ Inadequate myocardial suppression and spill over from the aortic root make confirmation of definite LMT uptake difficult. The authors recognized this and although attempts were made to compensate for myocardial and aortic spill over, the limited spatial resolution of human PET (6 mm) makes signal spill over and sampling errors more likely when imaging smaller vessels.

In a metanalysis of 10 RCTs, pioglitazone has been shown to reduce major adverse cardiovascular events, MI and stroke.^5^ Both insulin resistance and low-grade inflammation are associated with atherosclerotic progression and this group has previously shown pioglitazone to reduce atherosclerotic progression through a reduction in carotid inflammation as assessed by FDG PET/CT.^6^ However, despite insulin resistance and post prandial hyperglycaemia being associated with an elevated risk of cardiovascular events, there are no definitive data to suggest its reduction prevents them.

The author’s choice of tracer (FDG) is widely employed as a quantitative parameter of inflammatory cell activity, however, importantly lacks specificity for pro-inflammatory M1 macrophages. Other tracers, such as 68Ga-DOTATATE are specific for the somatostatin receptor 2 on M1 macrophages, have been used successfully in coronary disease and may be better suited.^7^ 11C-PK11195 is another inflammation targeted tracer which binds to the translocator receptor protein within the mitochondria of inflammatory cells. Uptake has been shown to correlate closely with TSPO binding sites in patients with carotid stenosis disease.^8^ Crucially, these tracers do not have the problems associated with non-specific uptake within the myocardium or glycolytically active tissue outside of atherosclerotic cardiovascular disease and we question whether FDG is the most suitable inflammation tracer to study in a diabetic population.

Reducing insulin resistance, systemic and atherosclerotic plaque inflammation are proven to reduce cardiovascular events. Hybrid PET/CT and PET/MR platforms now allow direct quantification of atherosclerotic plaque inflammatory activity and allow temporal tracking in response to systemic therapy. Tahara et al. have identified novel clinical variables which they believe may predict coronary inflammation in a population particularly vulnerable to cardiovascular events. We believe their findings should be interpreted with caution and would welcome a replicate study with a more specific inflammatory tracer unaffected by serum glucose (such as 68Ga-DOTATATE). Replication of the results would strengthen the case for a longitudinal cohort study to assess whether modification of these clinical variables translates to reduced coronary inflammation and event rates.
